# Fatal Renal Failure in a Spinal Cord Injury Patient with Vesicoureteric Reflux Who Underwent Repeated Ureteric Reimplantations Unsuccessfully: Treatment Should Focus on Abolition of High Intravesical Pressures rather than Surgical Correction of Reflux

**DOI:** 10.1155/2012/603715

**Published:** 2012-12-25

**Authors:** Subramanian Vaidyanathan, Bakul Soni, Kottarathil Abraham Abraham, Peter Hughes, Gurpreet Singh

**Affiliations:** ^1^Regional Spinal Injuries Centre, Southport and Formby District General Hospital, Town Lane, Southport PR8 6PN, UK; ^2^Department of Renal Medicine, Southport and Formby District General Hospital, Town Lane, Southport PR8 6PN, UK; ^3^Department of Radiology, Southport and Formby District General Hospital, Town Lane, Southport PR8 6PN, UK; ^4^Department of Urology, Southport and Formby District General Hospital, Town Lane, Southport PR8 6PN, UK

## Abstract

A 29-year-old man developed paraplegia at T-10 level due to road traffic accident in 1972. Both kidneys were normal and showed good function on intravenous urography. Division of external urethral sphincter was performed in 1973. In 1974, cystogram showed retrograde filling of left renal tract, which was hydronephrotic. Left ureteric reimplantation was performed. Following surgery, cystogram revealed marked retrograde filling of left renal tract as before. Penile sheath drainage was continued. In 1981, intravenous urography revealed bilateral severe hydronephrosis. Left ureteric reimplantation was performed again in 1983. Blood pressure was 220/140 mm Hg; this patient was prescribed atenolol. Cystogram showed gross left vesicoureteral reflux. Intermittent catheterisation was commenced in 2001. In 2007, proteinuria was 860 mg/day. This patient developed progressive renal failure and expired in 2012. In a spinal cord injury patient with vesicoureteral reflux, the treatment should focus on abolition of high intravesical pressures rather than surgical correction of vesicoureteric reflux. Detrusor hyperactivity and high intravesical pressures are the basic causes for vesicoureteral reflux in spinal cord injury patients. Therefore, it is important to manage spinal cord injury patients with neuropathic bladder by intermittent catheterisations along with antimuscarinic drug therapy in order to abolish high detrusor pressures and prevent vesicoureteral reflux. Angiotensin-converting enzyme inhibitors or angiotensin-receptor-blocking agents should be prescribed even in the absence of hypertension when a spinal cord injury patient develops vesicoureteral reflux and proteinuria.

## 1. Background

Spinal cord injury patients, who empty their bladder by increased abdominal pressure either by Valsalva or by Crede manoeuvres, are at risk for developing vesicoureteral reflux and hydronephrosis [1]. Lamid [2] noticed that the incidence of reflux was statistically higher in patients with complete spinal lesion than in those with incomplete neurological dysfunction; the incidence was also higher in individuals with an upper motor neuron lesion. Many spinal cord injury patients were on an indwelling Foley catheter at the time vesicoureteral reflux was detected, which indicated that free urinary drainage by a Foley catheter did not prevent occurrence of vesicoureteral reflux. Further, the indwelling Foley catheter proved ineffective for treatment of vesicoureteral reflux because in the long run, indwelling Foley catheter did not prevent progression of vesicoureteral reflux and did not protect the refluxing kidney from damage. Chartier Kastler and Ruffion [3] recommended that vesicoureteral reflux in spinal cord injury patients with neuropathic bladder should preferably be treated conservatively, as vesicoureteral reflux resolves in more than 90% of cases with effective reduction of intravesical pressures.

We report a spinal cord injury patient, who developed vesicoureteral reflux when he managed his bladder by sheath drainage. Ureteric reimplantation was performed twice but vesicoureteral reflux persisted. This patient developed hypertension and proteinuria followed by renal failure, to which he succumbed. The aim of this presentation is to emphasize the importance of preventing vesicoureteral reflux by reducing intravesical pressure. Effective reduction of intravesical pressure can be achieved in spinal cord injury patients by prescribing antimuscarinic drug and performing regular intermittent catheterisations. Abolition of high detrusor pressures should take precedence over surgical repair of vesicoureteral reflux by ureteric reimplantation in spinal injury patients. If intravesical pressures are not reduced, vesicoureteral reflux is likely to persist despite surgery as indeed happened to this spinal injury patient.

## 2. Case Presentation

A 29-year-old Caucasian male was involved in a road traffic accident in 1972 and sustained complete motor and sensory paralysis below the level of T-10. This patient had indwelling urethral catheter. Soon after injury, intravenous urography revealed functioning kidneys. Cystogram showed no vesicoureteral reflux. Division of external urethral sphincter was performed in 1973. Following surgery, this patient had penile sheath drainage. In 1974, cystogram revealed retrograde filling of left renal tract, which was hydronephrotic. Membranous urethrotomy was performed bilaterally. Left ureteric reimplantation was carried out through transverse suprapubic incision. There was marked cystitis with gross bladder trabeculations. Postoperatively, cystogram revealed marked retrograde filling of left renal tract as before. Intravenous urography revealed hydronephrotic changes in both kidneys. This patient continued to drain his bladder by condom catheter. In 1980, intravenous urography revealed marked hydronephrosis on left side and moderate hydronephrosis on right side. This patient was reviewed by a consultant urologist who noted deterioration of both kidneys over the years. However, intermittent catheterisation was not recommended. In 1981, intravenous urography revealed bilateral severe hydronephrosis. This patient developed recurrent urine infections. In 1983, cystogram revealed gross leftsided reflux. Renogram revealed poorly functioning left kidney. Indwelling urethral catheter drainage was established. In 1983, cystourethrogram revealed left vesicoureteral reflux. Left ureteric reimplantation was carried out. Submucosal tunnel was not possible so, new nipple was refashioned by cuff technique. Mucosal-to-mucosal anastamosis was performed. Blood pressure varied between 170/110 mm Hg and 220/140 mm Hg. This patient was prescribed Atenolol 50 mg a day increasing to 100 mg per day after three days. This patient again developed urine infection with *Pseudomonas*. He was prescribed Netilmicin 100 mg three times a day. Follow-up cystogram revealed gross left sided vesicoureteral reflux. This patient was prescribed Amikacin 500 mg twice a day for 48 hours. In 1985, this patient developed urine infections, which were treated by Cefotaxime for 5 to 7 days. This patient continued to drain his bladder by abdominal pressure, Crede manoeuvre, and straining. In 1986, this patient developed bilateral loin pain, nausea, lethargy, and loss of appetite. Urine was cloudy and smelly. He was prescribed Cefotaxime 1 gram twice a day for five days. In 1999, videourodynamics revealed abdominal pressure rising above 100 cm H_2_O. This patient was advised to perform intermittent catheterisations and avoid straining to pass urine. However, this patient was not able to perform more than two or three catheterisations per day. Therefore, indwelling urethral catheter drainage was established and the patient was prescribed modified-release oxybutynin 10 mg once a day. This patient developed bypassing of catheter, and the dose of oxybutynin was increased to 20 mg once a day. This patient continued to get recurrent urine infections. The indwelling catheter was removed and the patient started performing self-catheterisations five times a day and wore penile sheath. He was prescribed Tolterodine, but Tolterodine did not suit him. Therefore, this patient started taking modified-release oxybutynin, 10 mg once a day. Creatinine clearance, as estimated by analysis of 24-hour urine sample, revealed gradual decline: 50 mL/minute in February 2003; 44 mL/minute in November 2003; 31 mL/minute in May 2006. His medication included Oxybutynin, Atenolol, Bendroflumethiazide, Allopurinol, and prophylactic antibacterial for urine infection.

Isotope renogram with arsenide, performed in 2006, showed obstructive patterns in both kidneys. Divided renal function was calculated at 23% for the left kidney and 77% for the right kidney. In 2008, this patient developed severe urine infection and was prescribed Primaxin (Imipenem with Cilastatin). In 2009, this patient again developed severe urine infection and received Tazocin (Piperacillin with Tazobactam) intravenously. 

**Figure 1:**
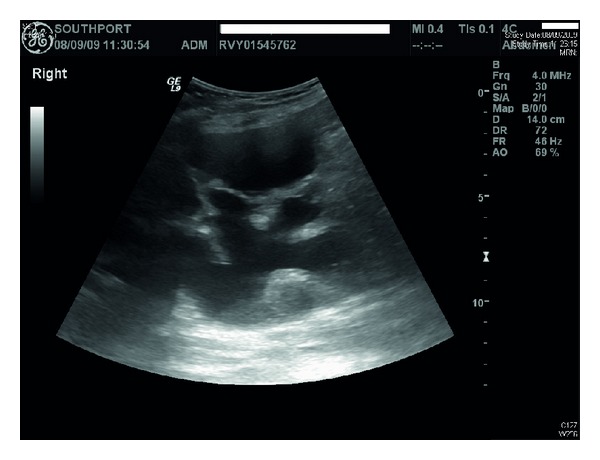
Ultrasound of urinary tract performed in 2009 revealed marked hydronephrosis of right kidney and bilateral renal cortical cysts.

**Figure 2:**
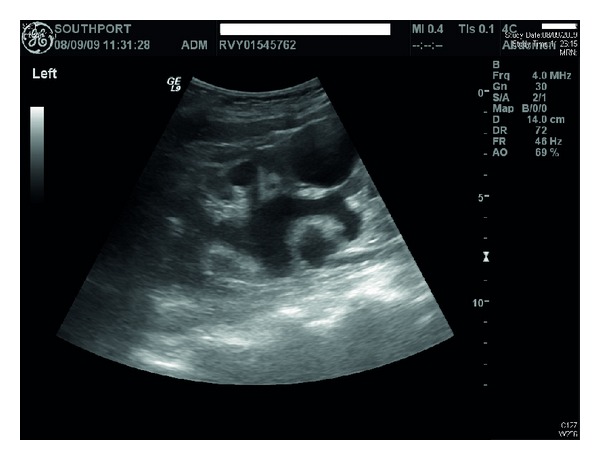
Ultrasound revealed moderate hydronephrosis of left kidney.

**Figure 3:**
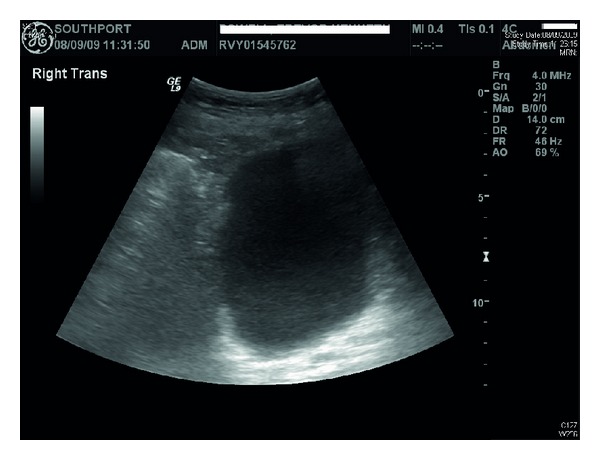
Ultrasound showed trabeculated bladder outline. There was no bladder mass.

**Figure 4:**
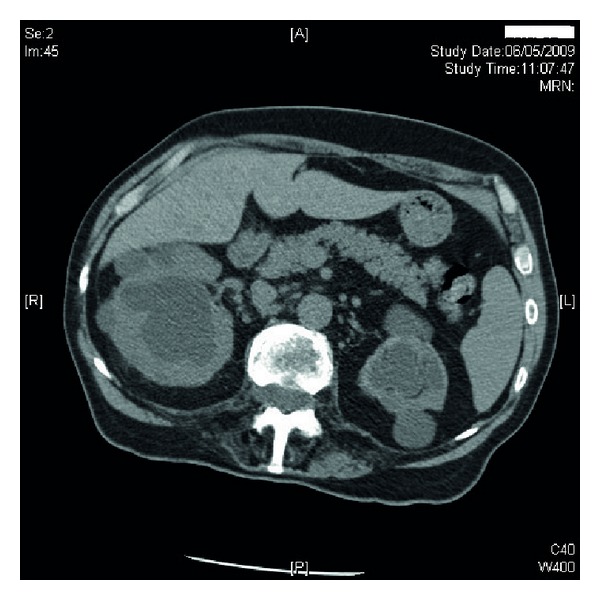
Computed tomography of abdomen revealed marked right hydronephrosis and mild-to-moderate left hydronephrosis. There were several large simple cortical cysts bilaterally, and there was also cortical thinning bilaterally.

Ultrasound of urinary tract revealed bilateral renal cortical cysts; marked hydronephrosis of right kidney (Figure 1); moderate hydronephrosis of left kidney (Figure 2); slightly trabeculated bladder outline (Figure 3); no renal or bladder mass. Computed tomography of abdomen was also performed, which confirmed marked right hydronephrosis and mild-to-moderate left hydronephrosis. There were several large simple cortical cysts bilaterally, and there was also cortical thinning bilaterally (Figure 4).

In 2011, 24-hour urine protein was 0.86 gram per day. Serum creatinine increased to 366 mmol/L. Serum albumin decreased to 12 g/L. Dietician advised low-phosphate, low-potassium diet. He was prescribed Furosemide 20 mg once a day and was asked to reduce fluid intake to 1.5 Litres a day maximum. Estimated glomerular filtration rate was 15 mL/minute. This patient developed paroxysmal atrial fibrillation due to withdrawal of atenolol. Brachiocephalic arteriovenous fistula was created in left arm. His condition deteriorated and he expired in February 2012.

## 3. Discussion

Ponce Diaz-Reixa and associates [4] studied 40 patients (80 renal units) with vesicoureteral reflux in neurogenic bladders and spinal cord injury, between March 1990 and November 2004. 77.5% of patients were males. Detrusor overactivity is found in 72.2% and detrusor-sphincter dyssynergia in 71.8%. Initial conservative treatment was done with indwelling catheter and anticholinergics. Complete remission was found only in 57.5% of renal units. It is essential to abolish detrusor hyperactivity and high intravesical pressures, which are the basic causes for vesicoureteral reflux [5]. The insertion of a Foley catheter understandably leads to an increase in both these factors acting, as it does, as a foreign body with the balloon of Foley catheter continuously stimulating the sensitive trigonal area. Lamid reiterated that high bladder pressure was one of the factors for vesicoureteral reflux formation in spinal cord injury patients and, therefore, recommended anticholinergic agent together with antibiotic prophylaxis in order to prevent further deterioration of the affected kidney [6]. Therefore, it is important to prevent vesicoureteral reflux by intermittent catheterisations along with antimuscarinic drug therapy.

An important aspect of preventing the progression of reflux nephropathy to end-stage renal failure is the treatment of hypertension and proteinuria, both of which are indicators of renal damage and contribute to ongoing deterioration of renal function [7]. Hypertension has been shown to affect the rate of decline of renal function; thus, controlling hypertension should be a significant goal for treatment of patients with vesicoureteral reflux.

In addition to control of hypertension, proteinuria has been correlated with the risk of chronic kidney disease in reflux nephropathy. Even mild proteinuria appears to be associated with increased risk of renal deterioration. El-Khatib and associates [8] showed an increased risk of deterioration of renal function for patients with >0.2 g per day of proteinuria with a progressively increased risk of deterioration for patients with >1 g per day of proteinuria. Proteinuria is a marker of renal damage and a perpetrator of kidney injury [9]. Reduction of proteinuria is associated with slowing the decline in kidney function. Proteinuria (>500 mg/day) is associated with increased mortality in persons with chronic spinal cord injury [10]. Apart from reflux nephropathy, risk factors for proteinuria in spinal cord injury patients are chronic indwelling bladder catheters, pressure ulcer, hypertension, older age, and longer duration of injury [11, 12].

Angiotensin-converting enzyme inhibitors may be able to slow the progression of renal deterioration associated with severe reflux nephropathy. There is evidence that in nondiabetic patients with renal parenchymal abnormalities, ACE inhibitors reduce proteinuria and may help to preserve renal function. ACE inhibitors or angiotensin receptor blocking agents should be the first choice for controlling hypertension and proteinuria and should be initiated early in the course of the disease. Furthermore, ACE inhibitors or angiotensin-receptor-blocking agents should be used even in the absence of hypertension when a patient has vesicoureteral reflux and proteinuria. Controlling hypertension and proteinuria should be considered standard maintenance therapy for those with vesicoureteral reflux and reflux nephropathy.

While reviewing our clinical practice [13], we realised that we had failed to prescribe angiotensin-converting enzyme inhibitor or angiotensin receptor blocking agent to some patients with reflux nephropathy and proteinuria. In a 69-year-old male with T-12 paraplegia, videourodynamics showed bilateral grade 3 vesicoureteral reflux and hyperreflexic contraction with detrusor pressures exceeding 100 cm of water. Intravenous urography showed marked bilateral hydronephrosis with bilateral cortical thinning. Blood test revealed creatinine level of 198 micromol/L. Twenty-four hours of urine collection showed creatinine clearance of 38 mL per minute, and urine protein of 2.38 grams. Bladder management was changed to indwelling urethral catheter drainage with oral oxybutynin therapy. Subsequently, ultrasound scan of the kidneys showed resolution of the hydronephrosis. There was, however, generalised cortical thinning, focal scarring of right upper pole, and reduction in the size of both kidneys. These changes were consistent with reflux nephropathy. 

Learning points from this case presentation are listed below.

- In a spinal cord injury patient with vesicoureteral reflux, the treatment should focus on abolition of high intravesical pressures rather than surgical procedures such as ureteric reimplantation. Vesicoureteral junction has a variable resistance to high pressure, if one gives way, the system is relatively decompressed and the opposite side is then not submitted to the same pressure and may remain intact. Unilateral vesicoureteral reflux is, therefore, not uncommon and should not be taken to mean that the problem lies with the vesicoureteral junction.
- Massive proteinuria is an uncommon development of vesicoureteral reflux and obstructive nephropathy. Spinal cord physician should consider the following differential diagnoses of proteinuria: (i) secondary focal segmental glomerulosclerosis, where loss of glomeruli occurs due to any insult, which causes the remaining glomeruli to leak protein at nephritic levels; (ii) primary glomerulonephritis such as membranous nephropathy. Treatment of proteinuria depends upon the underlying pathology: primary glomerulonephritides require immunosuppression, but patients with secondary glomerulosclerosis require management of the primary insult and other measures such as angiotensin-converting enzyme inhibitors.
- When a spinal cord injury patient with vesicoureteral reflux develops chronic kidney disease, preparing for dialysis and using drugs such as angiotensin-converting enzyme inhibitors are safe ways to control the proteinuria.
- Spinal cord physicians should work closely with renal physicians in order to provide the best care to patients with reflux nephropathy and most importantly, to slow the progression of chronic kidney disease in these patients. Frequent, informal discussions between spinal cord physicians, urologists, and renal physicians regarding patient management will help to improve the quality of patient care.Artificial barriers between hospitals, bureaucratic hurdles, and insistence on formal referral of patients do not help patients.

## 4. Conclusion

1. Detrusor hyperactivity and high intravesical pressures are the basic causes for vesicoureteral reflux in spinal cord injury patients. Therefore, it is important to manage spinal cord injury patients with neuropathic bladder by intermittent catheterisations along with antimuscarinic drug therapy in order to abolish high detrusor pressures and prevent vesicoureteral reflux.
2. Angiotensin-converting enzyme inhibitors or angiotensin receptor blocking agents should be prescribed even in the absence of hypertension when a spinal cord injury patient develops vesicoureteral reflux and proteinuria. Controlling hypertension and proteinuria should be considered standard maintenance therapy for those with vesicoureteral reflux and reflux nephropathy.
